# Aberrant hippocampus and amygdala morphology associated with cognitive deficits in schizophrenia

**DOI:** 10.3389/fncel.2023.1126577

**Published:** 2023-02-23

**Authors:** Bradley S. Peterson, Tejal Kaur, Siddhant Sawardekar, Tiziano Colibazzi, Xuejun Hao, Bruce E. Wexler, Ravi Bansal

**Affiliations:** ^1^Children’s Hospital Los Angeles, Department of Psychiatry at the Keck School of Medicine, University of Southern California, Los Angeles, CA, United States; ^2^Department of Psychiatry, Columbia College of Physicians and Surgeons, New York, NY, United States; ^3^Department of Psychiatry, Yale University School of Medicine, New Haven, CT, United States

**Keywords:** amygdala, schizophrenia, MRI, biomarker, endophenotype, working memory, hippocampus

## Abstract

**Background:**

Working memory deficits are thought to be a primary disturbance in schizophrenia. We aimed to identify differences in morphology of the hippocampus and amygdala in patients with schizophrenia compared with healthy controls (HCs), and in patients who were either neuropsychologically near normal (NPNN) or neuropsychologically impaired (NPI). Morphological disturbances in the same subfields of the hippocampus and amygdala, but of greater magnitude in those with NPI, would strengthen evidence for the centrality of these limbic regions and working memory deficits in the pathogenesis of schizophrenia.

**Methods:**

We acquired anatomical MRIs in 69 patients with schizophrenia (18 NPNN, 46 NPI) and 63 age-matched HC participants. We compared groups in hippocampus and amygdala surface morphologies and correlated morphological measures with clinical symptoms and working memory scores.

**Results:**

Schizophrenia was associated with inward deformations of the head and tail of the hippocampus, protrusion of the hippocampal body, and widespread inward deformations of the amygdala. In the same regions where we detected the effects of schizophrenia, morphological measures correlated positively with the severity of symptoms and inversely with working memory performance. Patients with NPI displayed a similar pattern of anatomical abnormality compared to patients with NPNN.

**Conclusion:**

Our findings indicate that anatomical abnormalities of the hippocampus relate to working memory performance and clinical symptoms in persons with schizophrenia. Moreover, NPNN and NPI patients may lie on a continuum of severity, both in terms of working memory abilities and altered brain structure, with NPI patients being more severe than NPNN patients in both domains.

## 1. Introduction

Symptoms of psychosis and cognitive deficits are the hallmarks of schizophrenia. Cognitive deficits in particular contribute to enduring functional impairments in persons with this illness (Green et al., 2004; Leifker et al., 2009). Working memory, one of the cognitive domains most affected in schizophrenia, involves the temporary storage of information within temporoparietal regions and the manipulation of that information by the dorsolateral prefrontal cortex (Baddeley, 1992; Barch, 2006; Lett et al., 2014). Evidence from animal (Yoon et al., 2008; Colgin, 2011; Gordon, 2011), fMRI (Karlsgodt et al., 2005; Anticevic et al., 2012), and electrophysiological (Benchenane et al., 2011) studies demonstrate that the maintenance and manipulation of information in working memory tasks relies on the integration of activity within the prefrontal cortex and hippocampus.

We previously reported that individuals with schizophrenia, compared to healthy control (HC) participants, have smaller volumes of white matter in regions underlying temporal, parietal, and sensorimotor cortices, and that these abnormalities increase in proportion to the degree of working memory deficits (Palmer et al., 1997; Colibazzi et al., 2013). Because 20–25% of patients across multiple studies display normal or near normal scores on tests of cognition that include working memory (Palmer et al., 1997; Weickert et al., 2000; Seaton et al., 2001), we further divided our patient group into those who were neuropsychologically near normal (NPNN) and those who were neuropsychologically impaired (NPI). We found that white matter abnormalities in NPI individuals were similar to, but more extensive than, those in the NPNN group, suggesting that NPI and NPNN test performance may represent a continuum of illness severity rather than differing neurobiological subtypes of illness.

Because corticolimbic circuits support working memory abilities (Karlsgodt et al., 2005; Benchenane et al., 2011; Colgin, 2011; Gordon, 2011; Anticevic et al., 2012), we now assess morphological characteristics of the hippocampus and amygdala in NPI and NPNN individuals relative to HC participants. NPNN persons could differ from NPI persons in either the magnitude or in the location of anatomical abnormalities within limbic regions of working memory circuits. Differing anatomical locations of disturbance would suggest differing underlying pathogenic mechanisms across groups. Similar anatomical locations in both NPI and NPNN individuals, but with differing magnitudes of anatomical disturbance, would suggest either similarities in pathogenic mechanism for these individuals or similarities in a common final pathway that contributes to working memory deficits.

Working memory deficits are prevalent in multiple neuropsychiatric disorders, but they are thought to be a primary disturbance in schizophrenia. Few studies (Colibazzi et al., 2013), however, have correlated working memory performance with disturbances in the same brain regions where we detect the effect of schizophrenia diagnosis, and therefore scant evidence directly supports the centrality of working memory in the pathogenesis of schizophrenia. However, if the degree of anatomical abnormalities identified in patients compared to HC participants are also associated with the severity of working memory deficits, that finding would support the centrality of working memory in the pathogenesis of schizophrenia.

Limbic regions, particularly the hippocampus, are thought to play a critical role in the pathogenesis of schizophrenia. Neuroimaging studies have detected abnormalities of the hippocampus in individuals with schizophrenia (Csernansky et al., 2002; Velakoulis et al., 2006; Vita et al., 2006; Mattai et al., 2011; Prestia et al., 2014), their unaffected family members (Tepest et al., 2003; Ho and Magnotta, 2010), and in persons at clinical high risk for schizophrenia (Velakoulis et al., 2006; Schobel et al., 2009), suggesting that these abnormalities predate illness and are not simply a consequence of being ill. They are therefore strong candidates for an endophenotype in schizophrenia (Gottesman and Gould, 2003). Abnormalities of the anterior hippocampus have been associated with positive symptoms and may predict the development of schizophrenia in putatively prodromal individuals (Schobel et al., 2009). Animal models support the theory that abnormal activity of the anterior hippocampus, with aberrant modulation from the basolateral amygdala, could contribute to abnormalities downstream within a circuit that includes the striatum and cortex (Sah et al., 2003; Lodge and Grace, 2008, 2011). In an animal model, these circuit-based abnormalities correlate with working memory performance (Li et al., 2011). However, to our knowledge, no prior studies in humans have reported correlations between structural abnormality of subregions of the hippocampus or amygdala with measures of working memory.

A previous study in our cohort of persons with schizophrenia reported differences between patient and HC groups in overall volumes of the hippocampus and amygdala. Deformation-based surface measures can detect variation within subregions of brain structures, however, and they therefore have greater sensitivity and regional specificity when assessing group differences in brain structure than total volume measures (Peterson, 2010). We therefore have applied these deformation-based methods to assess group differences within the hippocampus and amygdala and their correlations with working memory abilities. We hypothesized that we would detect significant morphological abnormalities of the hippocampus and amygdala in individuals with schizophrenia relative to HC participants, that these abnormalities in NPI participants would be similar to, but more extensive than, abnormalities in the NPNN participants, and that the magnitude of these abnormalities would correlate significantly with deficits of working memory and clinical symptoms of psychosis.

## 2. Methods and materials

We included 69 patients with schizophrenia (18 NPNN and 46 NPI) and 63 healthy age-matched control participants. Patients with schizophrenia were recruited from the Clinic at the Yale in New Haven, Connecticut. Control participants were randomly recruited from a list of 10,000 names purchased from a telemarketing company who were in the age range and lived in the same zip code area as patients with schizophrenia. Written informed consent was obtained for all the participants. Approval for the study was provided by the Human Investigation Committee of the Yale School of Medicine (New Haven) and by the institutional review board of the New York State Psychiatric Institute (New York). All patients met The Diagnostic and Statistical Manual of Mental Disorders (DSM)-IV criteria for a diagnosis of schizophrenia. Exclusion criteria for healthy participants included a history of axis I disorders, heavy substance use within the last 5 years, or neurological illness. Additional exclusion criteria for both groups included any previous seizure, head trauma with loss of consciousness, current or previous substance abuse, or intelligence quotient (IQ) below 70. Diagnoses were established through evaluation using the Schedule for Affective Disorders and Schizophrenia (Endicott and Spitzer, 1978) and a best-estimate consensus procedure that considered all available study materials and medical records. Symptom severities at the time of the study were assessed by doctoral level psychologists with established interrater reliability using the Positive and Negative Symptom Scale for Schizophrenia (Kay, 1990). All but three patients had been in treatment for over 5 years, most had been hospitalized more than three times (but none had been hospitalized within the 3 months preceding the study). None had abused substances for at least 60 days, and all had been taking their current medications for at least 30 days.

### 2.1. Definition of subgroups

As in our previous study, the patient group was divided into two subgroups based on neuropsychological performance on four serial position tasks (SPT), which assessed verbal and non-verbal working memory using four types of stimuli: words; easily named sounds, such as a ringing telephone; birdsongs; and snowflake designs (French et al., 2003). One group was considered NPI on the basis of overall scores on all four tests that were more than 1.0 SD below the mean of HC (NPI, *n* = 46). The second group was considered NPNN on the basis of overall working memory scores within 0.5 SD of the HC mean (NPNN, *n* = 18). These tests were selected for subgroup definition because patients with schizophrenia have been shown in previous studies to perform particularly poorly on these tasks, and because deficits in working memory in general, and verbal memory in particular, are among the most consistent and robust cognitive deficits in schizophrenia (Kay, 1990; French et al., 2003). We have previously validated the subgroups by comparing them to each other and to HC participants on the California Verbal Learning Test and a degraded stimulus continuous performance test, two tests widely used to demonstrate cognitive deficits in people with schizophrenia (LeDoux, 2007). In our sample of 76 patients, five were not classified as either NPNN or NPI because neuropsychological data were not available.

### 2.2. Clinical measures

Length of illness was a best-estimate judgment based on the patient’s report of the first appearance of symptoms or functional compromise related to the clinical features of schizophrenia (e.g., hallucinations, delusional thinking, paranoid fears, social withdrawal, decline in school, or work performance), first hospitalization, and history of illness as documented in reports related to initial hospitalizations. When insufficient information was available, no duration was assigned. Severity of illness was measured using the Positive and Negative Syndrome Scale (PANSS) (Lee et al., 2004). PANSS scores were available for 46 of the 69 patients in our sample. The two patient subgroups did not differ substantially in clinical symptoms (Table 1) or medication status (Table 2) (French et al., 2003).

**TABLE 1:** Demographic and clinical characteristics of study participants.

Characteristic	Patients (*N* = 69)	Healthy controls (*N* = 63)	df	*F*/*T* or χ^2^	*P*-value	NPI (*N* = 46)	NPNN (*N* = 18)	df	*F*/*T* or χ^2^	*P*-value
Age	42.3 (8.6)	32.9 (11.1)	131	29.4	**<0**.**001**	43.3 (8.9)	35.6 (8.2)	63	2.3	0.138
Sex (M:F)	(45:24)	(31:32)	1	3.46	0.063	28:18	15:3	1	2.96	0.085
Education	13.0 (2.4)	15.9 (4.7)	103	17.0	**0**.**001**	12.7 (2.3)	13.7 (2.3)	58	2.56	0.115
Minority/Caucasian (*N*)	27:42	11:52	1	7.54	**0**.**006**	17:29	7:11	1	0.021	0.886
Duration of illness (years)						18.4 (8.4)	18.0 (8.7)	59	0.025	0.874
Chlorpromazine equivalents (mg)						692.3 (626.5)	676.0 (471.6)	46	0.008	0.928
**PANSS scores**										
Positive						14.8 (5.7)	15.23 (6.0)	40	0.039	0.845
Negative						16.4 (6.6)	15.2 (5.1)	40	0.35	0.558
midrule General						31.7 (11.4)	31.97 (8.7)	40	0.005	0.943
Total						62.9 (20.9)	62.4 (16.4)	40	0.007	0.935
Working memory index						72.7 (3.8)	51.3 (10.2)	61	74.7	**<0**.**001**

*P*-values that are statistically significant at a significance level of 0.05 are indicated in bold.

**TABLE 2:** Psychotropic medication at the time of MRI scan.

Antipsychotic medication	NPI (*n* = 46)	NPNN (*n* = 18)
Any antipsychotic medication	43	16
Typical	17	3
Atypical	32	15
Typical + atypical	6	2
Missing data	2	0

### 2.3. MRI scanning and image pre-processing

MRI scans were obtained using a Siemens Sonata 1.5 Tesla scanner (Siemens, AG, Erlangen, Germany). Head positioning was standardized using canthomeatal landmarks. Anatomical images were acquired using a 3D MP-RAGE sequence (repetition time, 24 msec; echo time, 2.96 msec; 45° flip angle; 256 × 192 matrix; field of view, 30 cm; 2 excitations, slice thickness 1.2 mm; 124 contiguous slices encoded for sagittal slice reconstruction with voxel dimensions of 1.17 mm × 1.17 mm × 1.2 mm).

We corrected large-scale variations in image intensity using a validated algorithm developed at the Montreal Neurological Institute (Sled et al., 1998). We removed extracerebral tissues using an automated tool for extracting the brain (Shattuck and Leahy, 2002). Connecting dura was then removed manually on each slice in the sagittal view and checked in the orthogonal views. The brainstem was transected at the pontomedullary junction. We measured whole brain volume (WBV) for use as a covariate to control for global scaling effects in statistical analyses of conventional volumes. This measure included not only gray and white matter but also cerebrospinal fluid within the ventricles and cortical sulci to ensure the exclusion of any possible confound of age-related effects of tissue atrophy with this general measure of body scaling (Skullerud, 1985).

### 2.4. Hippocampus and amygdala morphology

Methods for defining the hippocampus and the amygdala followed previously published algorithms (Kates et al., 1997). The hippocampus and amygdala were traced manually on Sun Ultra 10 workstations using ANALYZE 8.0 software (Biomedical Imaging Resource, Mayo Foundation, Rochester, MN, USA), together with software developed in-house, while blind to participant characteristics and hemisphere (images were randomly flipped in the transverse plane before pre-processing and then reversed prior to statistical analysis) (Plessen et al., 2006). The rostral extent of the amygdala coincided with the most anterior section in which the anterior commissure crossed the midline. We determined the transition between the amygdala and hippocampus by a line connecting the inferior horn of the lateral ventricle with the amygdaloid sulcus or, when the sulcus was not obvious, with a straight horizontal line connecting the inferior horn of the lateral ventricle with the surface on the uncus (Watson et al., 1992). The most posterior section was the last section in which the crus of the fornix and the fimbria of the hippocampal formation could be delineated.

Our previously validated procedures for surface analysis (Bansal et al., 2007) were customized to accommodate independent analysis of the right and left hippocampus and amygdala. Briefly, a rigid-body similarity transformation with global scaling was used to register the entire brain of each participant with the template brain, thereby inherently controlling for WBV in all subsequent measurements. Each region (right and left hippocampus and amygdala) was then rigidly coregistered to the corresponding template region. This second transformation created a refined registration by which to compare surfaces of isolated regions. Each region was warped to the corresponding region of the template using a high-dimensional, non-rigid warping algorithm based on the principles of fluid-flow dynamics. Warping permitted point-to-point matching of the surfaces of the regions for each participant with the surfaces of the corresponding regions in the template brain. The high-dimensionally warped images were then unwarped to the refined affine registration, while maintaining the point-to-point correspondences established by the non-linear warping. These point correspondences permitted calculation of the signed Euclidean distance of each surface point from the corresponding point on the surface of the template region. Euclidean distances were compared statistically across groups to identify areas of protrusion or indention, from which we infer underlying increases or decreases in volume, respectively, along the surface of each amygdala and hippocampus.

Boundaries of subfields of the hippocampus and nuclei of the amygdala of the template brain were estimated using a modified version of a digital brain atlas (Chakravarty et al., 2006) with a widely used parcellation scheme and nomenclature (Hirai and Jones, 1989). The atlas was registered to the four template structures (right and left hippocampus and amygdala) using a 3-dimensional non-linear transformation based on voxel intensity. After smoothing this image manually, a simplified outline of the hippocampal subfields and amygdala nuclei, respectively, were overlaid on the respective template structures to aid localization of findings to individual subfields or nuclei.

### 2.5. Selection of the template brain

As we have done previously, we used a single representative brain as a template rather than an averaged brain from many participants, because a single brain has sharp borders at the cerebrospinal fluid (CSF)-gray matter or gray matter-white matter interface that improve the accuracy of registration (Plessen et al., 2006; Bansal et al., 2007; Amat et al., 2008). Averaging images for a template blurs these boundaries and increases registration errors that are important when distinguishing subtle effects across populations. In addition, precise surface morphometry requires a brain with smooth gray and white matter surfaces that are devoid of topological defects, which cannot be reconstructed by averaging brains from many individuals. This technique produces group differences that are independent of the specific template chosen.

To select the most appropriate, representative template brain for surface morphometry, we first selected as a preliminary template brain the HC participant whose age and overall brain size were nearest the average of all participants. The brains of the remaining participants were coregistered to this preliminary template, the point correspondences across their surfaces were identified as detailed above, and the distances of those points from the corresponding points on the preliminary template surface were calculated. The brain for which all points across its surface were closest, in the least squares sense, to the average of the distances across those points for the entire sample was selected as the final template because it was morphologically most representative of all brains in the cohort.

### 2.6. Statistical analyses

Analyses of conventional volumes were performed using SPSS version 17.0. We used a repeated measures analysis of variance over a spatial domain (the amygdala and hippocampus). The model included the within-participant factors of hemisphere with two levels (left and right) and region with two levels (amygdala and hippocampus). Diagnosis was a between-participants factor. Covariates included sex, age, and WBV. Similar analyses were repeated using only the patient group with diagnosis defined instead as NPI or NPNN. The model was determined through a procedure in which all main effects were forced into the model and higher-order terms were removed *via* backward stepwise selection with the constraint that the model was hierarchically well-formulated at each step.

For analyses of surface morphological features, we used linear regression at each voxel on the surface of each structure to compare across groups the signed Euclidean distances from points on the surfaces of the amygdala and hippocampus in each participant to the corresponding points on the surface of the respective reference structure. We applied a false discovery rate (FDR) correction threshold of *P* < 0.05 and color-coded probability values at each voxel across the surface of the reference structures. In all statistical analyses we covaried for age, age^2^, sex, and minority status (Caucasian or anything other than Caucasian).

## 3. Results

Socio-demographic and clinical characteristics of the groups are provided in Table 1. Patients compared with HCs were on average older, had fewer years of education, and were more likely to be an ethnic minority. We therefore included minority status and age, as well as age^2^ and sex, as covariates in all statistical analyses. We did not include years of education as a covariate, however, because the fewer year of education in patients with schizophrenia was a consequence of their illness, and therefore co-varying for it would risk eliminating the primary effect of interest, group differences in regional morphology between HC participants and patients with schizophrenia. The NPI and NPNN groups were similar in age, sex, education, and minority status, as well as in the severity of clinical symptoms, length of illness, and chlorpromazine equivalents of medication use (Table 2).

### 3.1. Conventional volumes

After adjusting for age, sex, minority status, and WBV, overall volumes of the hippocampus, and amygdala differed significantly between the patient and HC groups (F_127_ = 56.2; *p* = 0.0001) (Table 3). Overall volumes did not significantly differ between NPI and NPNN. We did not detect a significant interaction between either region (amygdala or hippocampus) and diagnosis, or between hemisphere and diagnosis. The mean ± SD WBV did not differ appreciably across diagnostic group (patients: 4,898 ± 53.5 cm^3^; controls: 4,821 ± 56.3 cm^3^; F_131_ = 0.87; *p* = 0.35), but did differ between NPI and NPNN participants (NPI: 4,908 ± 45.7 cm^3^; NPNN: 5,107 ± 74.3 cm^3^; F_63_ = 5.04; *p* = 0.029). Neither the severity of clinical symptoms nor the working memory index correlated with overall volumes.

**TABLE 3:** Overall volumes of the hippocampus and amygdala.

Conventional volumes	Patients (*n* = 69) vs. Controls (*n* = 63)*		NPI (*n* = 46) vs. NPNN (*n* = 18)	
Whole brain^a^ (cc)	4,898 ± 53.5	4,821 ± 56.3	4,908 ± 45.7	5,107 ± 74.3
Right hippocampus^b^ (mm^3^)	2,929.5 ± 47.8	3,212.5 ± 50.3	3,098.0 ± 102.5	2,943.4 ± 61.9
Left hippocampus^b^ (mm^3^)	2,945.3 ± 50.8	3,201.4 ± 53.5	3,068.9 ± 99.5	2,978.5 ± 60.1
Right amygdala^b^ (mm^3^)	1,561.0 ± 41.1	1,961.8 ± 43.2	1,564.5 ± 52.3	1,595.3 ± 31.6
Left amygdala^b^ (mm^3^)	1,553.0 ± 39.7	2,025.1 ± 41.9	1,562.6 ± 48.2	1,600.1 ± 29.1

^a^Mean value is corrected for age and sex.

^b^All mean values are corrected for age, sex, and whole brain volume (WBV). *Overall volumes of the hippocampus and amygdala differed significantly between the patient and HC groups (F_127_ = 56.2; *P* < 0.0001).

### 3.2. Surface analyses

**FIGURE 1:**
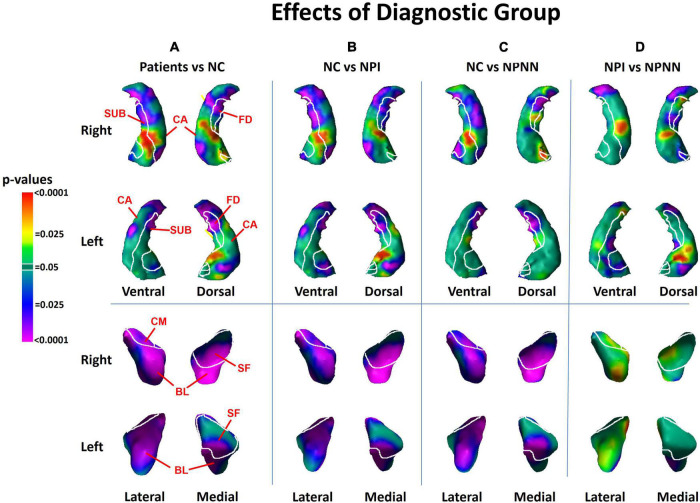
Main effects of diagnostic group on surface morphologic features. The right and left hippocampus are shown in their dorsal and ventral perspectives. The right and left amygdala are shown in their lateral and medical perspectives. The cytoarchitectonic map overlies the structures. At each point on the hippocampal amygdala surface, the statistical significance (probability values) of differences in surface morphology across risk-groups are color coded. The color bar indicates the color coding for *P*-values associated with the main effect of diagnostic group, with warmer colors (yellow and red) indicating protruding surfaces, presumably from larger underlying volumes, and cooler colors (blue and purple) indicating indented surfaces and presumably smaller underlying volumes in those regions. *P*-values are thresholded at *P* < 0.05 after correction for multiple comparisons using false discovery rate (FDR). The statistical model included the main effect of diagnostic group, and the covariates of age, age^2^, sex, and minority status. Statistical maps for the differences in surface morphology of the hippocampus and amygdala are shown for **(A)** individuals with schizophrenia vs. healthy control (HC); **(B)** neuropsychologically impaired (NPI) vs. HC; **(C)** neuropsychologically near normal (NPNN) vs. HC; and **(D)** NPI vs. NPNN. CA, cornu ammonis; FD, fascia dentata; SUB, subiculum; SF, superficial nucleus; CM, centromedial nucleus; BL, basolateral nucleus.

**FIGURE 2:**
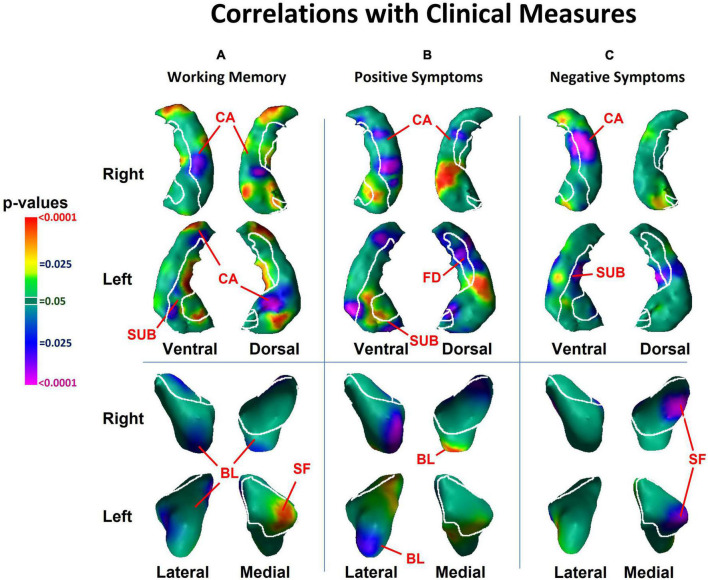
Associations with surface morphology of the hippocampus and amygdala for clinical measures of **(A)** working memory performance, **(B)** positive psychosis symptom severity, and **(C)** negative psychosis symptom severity. Here, working memory scores are inverted so that higher scores indicate greater deficits in working memory. Higher values of positive and negative symptoms indicate greater clinical symptoms. The cytoarchitectonic map overlies the structures. CA, cornu ammonis; FD, fascia dentata; SUB, subiculum; SF, superficial nucleus; CM, centromedial nucleus; BL, basolateral nucleus.

We detected significant diagnosis effects as inward deformations in the head and tail of the hippocampus, protrusion of the hippocampal body, and diffuse inward deformations of the amygdala (Figure 1A). We detected similar patterns of group differences when we compared NPI and NPNN separately to HC participants (Figures 1B, C). However, the NPI compared to NPNN group had greater inward deformations of the head and tail, as well as greater protrusion of the body of the hippocampus (Figure 1D). The NPI compared to NPNN group also had a greater indentation in the ventral aspect of the left amygdala, but an attenuated indentation in the ventral posterior aspect of the left amygdala (Figure 1D). We detected several significant correlations of surface measures with symptom severity and working memory performance within the schizophrenia patients. Worse performance on working memory tasks accompanied inward deformation of the head and tail, and protrusion of the body, of the hippocampus (Figure 2A) in similar regions where we detected the effects of diagnosis (patient group compared to HC).

More severe positive symptoms accompanied inward deformation of the head and tail and protrusion of the body of the hippocampus, and more severe negative symptoms accompanied inward deformation of the tail (Figure 2B), all in regions close to those where we detected the effects of diagnosis. We detected an inverse association of both positive symptoms with surface measures at the lateral aspect of the basolateral nucleus (BL) of the amygdala (Figure 2B), and of negative symptoms with surface measures at the medial aspect of the superficial nucleus (Figure 2C), such that greater severity of positive and negative symptoms accompanied larger inward deformations. We also detected a positive association of working memory performance with surface measures of the medial aspect of the superficial nucleus. None of the cognitive or clinical associations with the surface of the amygdala overlapped with regions where we detected the effect of diagnosis.

**FIGURE 3:**
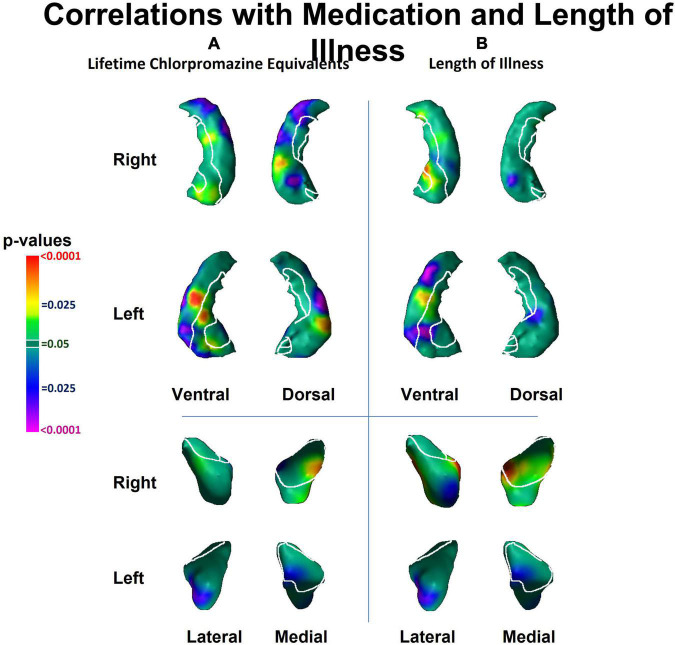
Associations of lifetime chlorpromazine equivalents and of length of illness with surface morphology of the hippocampus and amygdala. Statistical maps for the association of panel **(A)** lifetime chlorpromazine equivalents and **(B)** length of illness with surface morphological measures (Euclidian distances) of the hippocampus and amygdala. The effects of medication and lifetime illness on surface measures overlapped with the regions of the hippocampus and the amygdala where we detected diagnosis effects (Figure 1).

The effects of medication use (chlorpromazine equivalents of antipsychotic medication at time of MRI scan) and length of illness both consisted of predominant inward deformations of the head, body and tail of the hippocampus bilaterally and of the left lateral amygdala (Figure 3). The effect of medication and chronicity of illness overlapped with the regions where we detected diagnosis effects. However, the effect of diagnosis on the surface measures of the hippocampus and amygdala remained similar even after including medication and chronicity of illness as covariates in our statistical analyses. Furthermore, within the hippocampus, the pattern of group differences when we compared NPI to NPNN was similar to the main effect of diagnosis (patients vs. HC) (Figure 1D), even though NPI and NPNN did not differ in length of illness or chlorpromazine equivalents (Tables 1, 2).

## 4. Discussion

This is, to our knowledge, the first study to assess the associations of morphological abnormalities in hippocampus and amygdala subfields with working memory deficits in persons who have schizophrenia. Our findings collectively and strongly suggest that both the morphological disturbances and working memory deficits are tightly linked and are central to the pathogenesis of schizophrenia. We demonstrated that individuals with schizophrenia compared to HCs had inward deformations in the head and tail of the hippocampus, protrusion of the hippocampal body, and diffuse inward deformations of the amygdala. The locations of abnormalities were similar in NPNN and NPI participants, despite the NPNN participants having relatively normal cognitive abilities. However, the NPI participants had a greater magnitude of morphological abnormalities compared to NPNN participants. We previously reported that the NPI and NPNN groups displayed a similar pattern of white matter abnormalities within the cortex, but with NPI participants showing greater magnitude of structural abnormality as well as greater degree of working memory deficits (Colibazzi et al., 2013). Taken together with these previous findings, our current findings suggest that NPI and NPNN participants lie on a continuum of severity in terms of both the magnitude of neuroanatomical disturbance and cognitive ability, with those who are NPI being more severe in both domains.

We also found that the magnitude of clinical symptoms and working memory deficits associated positively with surface deformations in similar locations within the hippocampus where we identified group differences between patients and HC. Worse working memory performance accompanied inward deformations of the head and tail, and outward deformation of the body, of the hippocampus. Similarly, more severe positive symptoms accompanied inward deformation of the head and tail and protrusion of the body of the hippocampus, and more severe negative symptoms accompanied inward deformation of the tail. Most of the associations of symptom measures and working memory performance with surface measures of the hippocampus occurred in regions where we detected group differences between patients and HCs. Within the amygdala, greater severity of positive symptoms accompanied larger inward deformations of the lateral aspect of the BL, and negative symptoms accompanied larger inward deformations of the medial aspect of the superficial nucleus. Because the associations of clinical and neurocognitive measures with surface measures of the hippocampus were located in similar portions of the hippocampus where we detected differences between patients and HC, our findings suggest that an increasing magnitude of neuroanatomical abnormalities within the hippocampus may contribute to the cognitive deficits and clinical symptoms of schizophrenia. However, future studies that include individuals at high-risk for psychosis are necessary to test whether these neuroanatomical abnormalities precede and likely contribute to the genesis of frank psychosis, or whether they are a consequence of psychotic illness or its treatment. Furthermore, even if NPI and NPNN share similar structural abnormalities, it is possible that these abnormalities are the common downstream consequences of differing etiologic pathways.

### 4.1. Hippocampus subfields

We detected inward deformations in the head and tail, and protrusion in the body, of the hippocampus in persons with schizophrenia compared to HCs. The magnitude of positive symptoms and working memory deficits associated significantly with the structural abnormalities we detected along the head, body and tail of the hippocampus in the cornu ammonis (CA), fascia dentata (FD), and subiculum (SUB) subfields, whereas the magnitude of negative symptoms associated significantly with the structural abnormalities along the body and tail of the hippocampus in the CA and FD subfields.

Each hippocampus subfield participates in a distinct component of learning and memory, with the SUB supporting memory retrieval, the FD and CA3 classifying those memories into patterns (“pattern separation”), the CA3 completing the recognition of those patterns in relation to stored memories (“pattern completion”), and the CA1 integrating these inputs within the hippocampus (Small et al., 2011). Disturbances in these components of learning and memory could lead to errors when categorizing stimuli, thereby generating delusional or hallucinatory symptoms (Corlett et al., 2009; Tamminga et al., 2010). Whereas positive symptoms and working memory performance accompanied anatomical abnormalities of the FD, CA, and SUB, the specificity of negative symptoms to FD and CA, but not to the SUB, could suggest that negative symptoms relate more to difficulties in recognizing and completing patterns than to disturbances in memory retrieval.

Our finding that negative and positive symptoms and working memory performance were associated with distinct subregions of the hippocampus likely also reflects the regional distinctions in function along the longitudinal axis of the hippocampus. The anterior portion projects prominently to medial prefrontal and orbitofrontal cortices, as well as to the amygdala, and is thought to support the emotional content of cognitive processes (Fanselow and Dong, 2010). The posterior portion of the hippocampus projects to the anterior cingulate, posterior cingulate, and retrosplenial cortices, which together support primarily the non-emotional components of memory (Bast and Feldon, 2003; Fanselow and Dong, 2010). Our findings that working memory performance and positive symptoms accompany surface abnormalities of both the head and tail, suggest that emotional and cognitive processes likely contribute to both working memory performance and positive symptoms, a conclusion supported by prior studies (Yang et al., 2013). In contrast, negative symptoms correlated with surface measures in the tail and body, perhaps because negative symptoms may associate with primarily cognitive, rather than emotional components of information processing. These and other interpretations are testable hypotheses for future studies that are informed by our neuroanatomical findings.

Our finding that hippocampus dysfunction accompanies symptoms of psychosis and neurocognitive deficits is consistent with prior studies. Both animal models and human research suggest that hypermetabolism of the anterior hippocampus may drive downstream disturbances in dopamine release in the striatum (Lodge and Grace, 2008, 2011) that are thought to generate symptoms of psychosis and contribute to cognitive deficits (Seaton et al., 2001; Howes et al., 2011). Increased cerebral blood volume (CBV) in the anterior hippocampus in CA1 reportedly associates with positive symptoms in both individuals with schizophrenia and those prodromal for psychosis (Schobel et al., 2009). Human studies and animal models suggest that the initial increase in CBV in CA1 spreads to the SUB during the onset of psychosis and appears to predate hippocampal atrophy (Schobel et al., 2013). Studies using surface measures have also detected inward deformations of the hippocampal head (Csernansky et al., 2002; Tepest et al., 2003; Narr et al., 2004; Ho and Magnotta, 2010; Johnson et al., 2013) and tail (Lee et al., 2004; Styner et al., 2004), as well as protrusion of the CA regions (Mamah et al., 2012) in individuals with schizophrenia, and one of these studies has correlated these findings with the severity of positive symptoms (Zierhut et al., 2013). Our study extends prior findings because we detected associations of working memory and clinical symptoms with surface measures in similar regions of the hippocampus where we detected group differences between patients and HC, suggesting that the neural correlates in the hippocampus of working memory, and of positive and negative symptoms, likely contribute to the pathogenesis of schizophrenia.

### 4.2. Amygdala subfields

We detected indentations throughout the amygdala in individuals with schizophrenia, in regions overlying all the major nuclei. The locations of abnormalities were similar in NPNN and NPI participants, but the NPI, compared to NPNN, group had larger indentations in the ventral aspect of the left amygdala, and attenuated indentation in the ventral posterior aspect of the left amygdala. In contrast to the diffuse abnormalities detected as group differences, we also detected associations with symptom measures in specific subregions. Surface measures of the basolateral nuclei associated significantly with positive symptoms, and the superficial nuclei associated significantly with negative symptoms, such that greater symptom severity accompanied greater magnitude of inward deformation.

Together, the basolateral and superficial nuclei subserve the comprehension of social context and the modulation of affective states with goal-directed actions. The basolateral nuclei project reciprocally to widespread cortical and subcortical regions that receive sensory input for social cues and that govern behaviors guided by these sensory stimuli (Amaral, 2002; Sah et al., 2003; LeDoux, 2007; Amaral et al., 2008). The basolateral nuclei of the amygdala and CA at the head of the hippocampus project reciprocally to one another (French et al., 2003). Dysfunction of the basolateral nuclei has been theorized to contribute to GABAergic dysfunction of the hippocampus (French et al., 2003; Sesack and Grace, 2010) and could modulate the emergence of positive symptoms. The superficial nuclei selectively extracts the social from non-social value of incoming sensory cues, particularly in the processing of facial expressions (Bzdok et al., 2012), abnormalities in which could lead to negative psychotic symptoms. Impairments in these capacities could produce social and functional deficits that contribute to the clinical symptoms that often characterize schizophrenia. Surface measures of subregions of the amygdala associated significantly with clinical symptoms and cognitive deficits, even though these same regions did not overlap with the subregions where we detected differences between patient and control groups, suggesting perhaps that these morphological features may modulate the severity of clinical symptoms and cognitive deficits but are unlikely to be central in causing schizophrenia.

### 4.3. Limitations and future directions

The primary limitation of our study is that we cannot distinguish the causes from consequences of illness in a sample of already ill individuals (Horga et al., 2014). Moreover, the effects of length of illness and lifetime medication on surface measures overlapped with the regions in which we detected the effect of diagnosis, suggesting that the effects of chronic illness and medication could contribute to our findings. However, because we continue to detect an effect of diagnosis when co-varying for medication and illness chronicity, our findings likely are present above and beyond simply the effects of chronic illness or stress. Moreover, we detected similar group differences between NPNN and NPI participants as we did between patients and HC, even though length of illness and chlorpromazine equivalents did not differ between NPNN and NPI. Therefore, our findings suggest that the pattern of inward deformation in the head and tail and protrusion in the body that we detected as an effect of diagnosis is not simply a consequence of illness. Furthermore, studies using surface analyses that include unaffected siblings of persons with schizophrenia also detect inward deformations of the anterior hippocampus (Tepest et al., 2003; Ho and Magnotta, 2010; Johnson et al., 2013), suggesting that surface deformations are not simply due to illness chronicity or medication exposure, but could be a vulnerability marker or endophenotype for schizophrenia. Subsequent longitudinal studies correlating surface measures with clinical and cognitive domains in unaffected individuals who are at high-risk for schizophrenia are necessary to distinguish whether our findings predate illness and contribute to generation of symptoms or whether they represent the consequence of illness. Future studies are also necessary to understand whether the cognitive deficits of NPNN and NPI participants precede illness or arise during illness or its treatment, and whether those cognitive deficits correspond longitudinally to anatomical changes in the brain.

## Data availability statement

The raw data supporting the conclusions of this article will be made available by the authors, without undue reservation.

## Ethics statement

The studies involving human participants were reviewed and approved by the Yale School of Medicine and the New York State Psychiatric Institute. The patients/participants provided their written informed consent to participate in this study.

## Author contributions

BP, BW, and RB: conceptualization and methodology. RB and XH: software. BP, SS, XH, and RB: image processing. BP, TK, TC, BW, and RB: statistical analysis. BP and BW: assessments and funding acquisition. BP, TK, BW, and RB: writing – original draft preparation. BP, TK, TC, BW, XH, and RB: writing – review and editing. All authors contributed to the article and approved the submitted version.

## References

[B1] Amaral D. G. (2002). The primate amygdala and the neurobiology of social behavior: Implications for understanding social anxiety. *Biol. Psychiatry* 51 11–17. 11801227 10.1016/s0006-3223(01)01307-5

[B2] Amaral D. G. Schumann C. M. Nordahl C. W. (2008). Neuroanatomy of autism. *Trends Neurosci.* 31 137–145. 10.1016/j.tins.2007.12.005 18258309

[B3] Amat J. A. Bansal R. Whiteman R. Haggerty R. Royal J. Peterson B. S. (2008). Correlates of intellectual ability with morphology of the hippocampus and amygdala in healthy adults. *Brain Cogn.* 66 105–114. 10.1016/j.bandc.2007.05.009 17651879 PMC2291291

[B4] Anticevic A. Repovs G. Krystal J. H. Barch D. M. (2012). A broken filter: Prefrontal functional connectivity abnormalities in schizophrenia during working memory interference. *Schizophr. Res.* 141 8–14. 10.1016/j.schres.2012.07.007 22863548 PMC3879404

[B5] Baddeley A. (1992). Working memory. *Science* 255 556–559. 1736359 10.1126/science.1736359

[B6] Bansal R. Staib L. H. Xu D. Zhu H. Peterson B. S. (2007). Statistical analyses of brain surfaces using Gaussian random fields on 2-D manifolds. *IEEE Trans. Med. Imaging* 26 46–57. 10.1109/TMI.2006.884187 17243583 PMC2366175

[B7] Barch D. M. (2006). What can research on schizophrenia tell us about the cognitive neuroscience of working memory? *Neuroscience* 139 73–84. 10.1016/j.neuroscience.2005.09.013 16300901

[B8] Bast T. Feldon J. (2003). Hippocampal modulation of sensorimotor processes. *Prog. Neurobiol.* 70 319–345. 12963091 10.1016/s0301-0082(03)00112-6

[B9] Benchenane K. Tiesinga P. H. Battaglia F. P. (2011). Oscillations in the prefrontal cortex: A gateway to memory and attention. *Curr. Opin. Neurobiol.* 21 475–485. 10.1016/j.conb.2011.01.004 21429736

[B10] Bzdok D. Laird A. R. Zilles K. Fox P. T. Eickhoff S. B. (2012). An investigation of the structural, connectional, and functional subspecialization in the human amygdala. *Hum. Brain Mapp.* 34 3247–3266. 10.1002/hbm.22138 22806915 PMC4801486

[B11] Chakravarty M. M. Bertrand G. Hodge C. P. Sadikot A. F. Collins D. L. (2006). The creation of a brain atlas for image guided neurosurgery using serial histological data. *Neuroimage* 30 359–376. 10.1016/j.neuroimage.2005.09.041 16406816

[B12] Colgin L. L. (2011). Oscillations and hippocampal-prefrontal synchrony. *Curr. Opin. Neurobiol.* 21 467–474. 10.1016/j.conb.2011.04.006 21571522 PMC3578407

[B13] Colibazzi T. Wexler B. E. Bansal R. Hao X. Liu J. Sanchez-Pena J. (2013). Anatomical abnormalities in gray and white matter of the cortical surface in persons with schizophrenia. *PLoS One* 8:e55783. 10.1371/journal.pone.0055783 23418459 PMC3572102

[B14] Corlett P. R. Krystal J. H. Taylor J. R. Fletcher P. C. (2009). Why do delusions persist? *Front. Hum. Neurosci.* 3:12. 10.3389/neuro.09.012.2009 19636384 PMC2713737

[B15] Csernansky J. G. Wang L. Jones D. Rastogi-Cruz D. Posener J. A. Heydebrand G. (2002). Hippocampal deformities in schizophrenia characterized by high dimensional brain mapping. *Am. J. Psychiatry* 159 2000–2006. 12450948 10.1176/appi.ajp.159.12.2000

[B16] Endicott J. Spitzer R. L. (1978). A diagnostic interview: The schedule for affective disorders and schizophrenia. *Arch. Gen. Psychiatry* 35 837–844. 678037 10.1001/archpsyc.1978.01770310043002

[B17] Fanselow M. S. Dong H. W. (2010). Are the dorsal and ventral hippocampus functionally distinct structures? *Neuron* 65 7–19. 10.1016/j.neuron.2009.11.031 20152109 PMC2822727

[B18] French S. J. Hailstone J. C. Totterdell S. (2003). Basolateral amygdala efferents to the ventral subiculum preferentially innervate pyramidal cell dendritic spines. *Brain Res.* 981 160–167. 10.1016/s0006-8993(03)03017-8 12885437

[B19] Gordon J. A. (2011). Oscillations and hippocampal-prefrontal synchrony. *Curr. Opin. Neurobiol.* 21 486–491. 10.1016/j.conb.2011.02.012 21470846 PMC3138872

[B20] Gottesman I. I. Gould T. D. (2003). The endophenotype concept in psychiatry: Etymology and strategic intentions. *Am. J. Psychiatry* 160 636–645. 12668349 10.1176/appi.ajp.160.4.636

[B21] Green M. F. Kern R. S. Heaton R. K. (2004). Longitudinal studies of cognition and functional outcome in schizophrenia: Implications for MATRICS. *Schizophr. Res.* 72 41–51. 10.1016/j.schres.2004.09.009 15531406

[B22] Hirai T. Jones E. G. (1989). A new parcellation of the human thalamus on the basis of histochemical staining. *Brain Res. Brain Res. Rev.* 14 1–34. 10.1016/0165-0173(89)90007-6 2720229

[B23] Ho B. C. Magnotta V. (2010). Hippocampal volume deficits and shape deformities in young biological relatives of schizophrenia probands. *Neuroimage* 49 3385–3393. 10.1016/j.neuroimage.2009.11.033 19941961 PMC2818551

[B24] Horga G. Kaur T. Peterson B. S. (2014). Annual research review: Current limitations and future directions in MRI studies of child- and adult-onset developmental psychopathologies. *J. Child Psychol. Psychiatry* 55 659–680. 10.1111/jcpp.12185 24438507 PMC4029914

[B25] Howes O. Bose S. Turkheimer F. Valli I. Egerton A. Stahl D. (2011). Progressive increase in striatal dopamine synthesis capacity as patients develop psychosis: A PET study. *Mol. Psychiatry* 16 885–886. 10.1038/mp.2011.20 21358709 PMC3662873

[B26] Johnson S. L. Wang L. Alpert K. I. Greenstein D. Clasen L. Lalonde F. (2013). Hippocampal shape abnormalities of patients with childhood-onset schizophrenia and their unaffected siblings. *J. Am. Acad. Child Adolesc. Psychiatry* 52 527–536.e2. 10.1016/j.jaac.2013.02.003 23622854 PMC3812431

[B27] Karlsgodt K. H. Shirinyan D. van Erp T. G. Cohen M. S. Cannon T. D. (2005). Hippocampal activations during encoding and retrieval in a verbal working memory paradigm. *Neuroimage* 25 1224–1231. 10.1016/j.neuroimage.2005.01.038 15850740

[B28] Kates W. R. Abrams M. T. Kaufmann W. E. Breiter S. N. Reiss A. L. (1997). Reliability and validity of MRI measurement of the amygdala and Hippocampus in children with fragile X syndrome. *Psychiatry Res.* 75 31–48. 10.1016/s0925-4927(97)00019-x 9287372

[B29] Kay S. R. (1990). Positive-negative symptom assessment in schizophrenia: Psychometric issues and scale comparison. *Psychiatr. Q.* 61 163–178. 10.1007/BF01064966 2075220

[B30] LeDoux J. (2007). The amygdala. *Curr. Biol.* 17 R868–R874. 10.1016/j.cub.2007.08.005 17956742

[B31] Lee J. M. Kim S. H. Jang D. P. Ha T. H. Kim J. J. Kim I. Y. (2004). Deformable model with surface registration for hippocampal shape deformity analysis in schizophrenia. *Neuroimage* 22 831–840. 10.1016/j.neuroimage.2004.02.004 15193612

[B32] Leifker F. R. Bowie C. R. Harvey P. D. (2009). Determinants of everyday outcomes in schizophrenia: The influences of cognitive impairment, functional capacity, and symptoms. *Schizophr. Res.* 115 82–87. 10.1016/j.schres.2009.09.004 19775869

[B33] Lett T. A. Voineskos A. N. Kennedy J. L. Levine B. Daskalakis Z. J. (2014). Treating working memory deficits in schizophrenia: A review of the neurobiology. *Biol. Psychiatry* 75 361–370. 10.1016/j.biopsych.2013.07.026 24011822

[B34] Li Y. C. Kellendonk C. Simpson E. H. Kandel E. R. Gao W. J. (2011). D2 receptor overexpression in the striatum leads to a deficit in inhibitory transmission and dopamine sensitivity in mouse prefrontal cortex. *Proc. Natl. Acad. Sci. U.S.A.* 108 12107–12112. 10.1073/pnas.1109718108 21730148 PMC3141950

[B35] Lodge D. J. Grace A. A. (2008). Hippocampal dysfunction and disruption of dopamine system regulation in an animal model of schizophrenia. *Neurotox. Res.* 14 97–104. 10.1007/BF03033801 19073417 PMC2879641

[B36] Lodge D. J. Grace A. A. (2011). Hippocampal dysregulation of dopamine system function and the pathophysiology of schizophrenia. *Trends Pharmacol. Sci.* 32 507–513. 10.1016/j.tips.2011.05.001 21700346 PMC3159688

[B37] Mamah D. Harms M. P. Barch D. Styner M. Lieberman J. A. Wang L. (2012). Hippocampal shape and volume changes with antipsychotics in early stage psychotic illness. *Front. Psychiatry* 3:96. 10.3389/fpsyt.2012.00096 23162479 PMC3495266

[B38] Mattai A. Hosanagar A. Weisinger B. Greenstein D. Stidd R. Clasen L. (2011). Hippocampal volume development in healthy siblings of childhood-onset schizophrenia patients. *Am. J. Psychiatry* 168 427–435. 10.1176/appi.ajp.2010.10050681 21245087 PMC3289129

[B39] Narr K. L. Thompson P. M. Szeszko P. Robinson D. Jang S. Woods R. P. (2004). Regional specificity of hippocampal volume reductions in first-episode schizophrenia. *Neuroimage* 21 1563–1575. 10.1016/j.neuroimage.2003.11.011 15050580

[B40] Palmer B. W. Heaton R. K. Paulsen J. S. Kuck J. Braff D. Harris M. J. (1997). Is it possible to be schizophrenic yet neuropsychologically normal? *Neuropsychology* 11 437–446. 9223148 10.1037//0894-4105.11.3.437

[B41] Peterson B. S. (2010). Form determines function: New methods for identifying the neuroanatomical loci of circuit-based disturbances in childhood disorders. *J. Am. Acad. Child Adolesc. Psychiatry* 49 533–538. 10.1016/j.jaac.2010.03.010 20494263 PMC2891511

[B42] Plessen K. J. Bansal R. Zhu H. Whiteman R. Amat J. Quackenbush G. A. (2006). Hippocampus and amygdala morphology in attention-deficit/hyperactivity disorder. *Arch. Gen. Psychiatry* 63 795–807. 16818869 10.1001/archpsyc.63.7.795 PMC2367150

[B43] Prestia A. Cavedo E. Boccardi M. Muscio C. Adorni A. Geroldi C. (2014). Hippocampal and amygdalar local structural differences in elderly patients with schizophrenia. *Am. J. Geriatr. Psychiatry* 23 47–58. 10.1016/j.jagp.2014.01.006 24534522 PMC4382088

[B44] Sah P. Faber E. S. Lopez De Armentia M. Power J. (2003). The amygdaloid complex: Anatomy and physiology. *Physiol. Rev.* 83 803–834. 10.1152/physrev.00002.2003 12843409

[B45] Schobel S. A. Chaudhury N. H. Khan U. A. Paniagua B. Styner M. A. Asllani I. (2013). Imaging patients with psychosis and a mouse model establishes a spreading pattern of hippocampal dysfunction and implicates glutamate as a driver. *Neuron* 78 81–93. 10.1016/j.neuron.2013.02.011 23583108 PMC3966570

[B46] Schobel S. A. Lewandowski N. M. Corcoran C. M. Moore H. Brown T. Malaspina D. (2009). Differential targeting of the CA1 subfield of the hippocampal formation by schizophrenia and related psychotic disorders. *Arch. Gen. Psychiatry* 66 938–946. 10.1001/archgenpsychiatry.2009.115 19736350 PMC2797730

[B47] Seaton B. E. Goldstein G. Allen D. N. (2001). Sources of heterogeneity in schizophrenia: The role of neuropsychological functioning. *Neuropsychol. Rev.* 11 45–67. 11392562 10.1023/a:1009013718684

[B48] Sesack S. R. Grace A. A. (2010). Cortico-Basal Ganglia reward network: Microcircuitry. *Neuropsychopharmacology* 35 27–47. 10.1038/npp.2009.93 19675534 PMC2879005

[B49] Shattuck D. W. Leahy R. M. (2002). BrainSuite: An automated cortical surface identification tool. *Med. Image Anal.* 6 129–142. 10.1016/s1361-8415(02)00054-3 12045000

[B50] Skullerud K. (1985). Variations in the size of the human brain. Influence of age, sex, body length, body mass index, alcoholism, Alzheimer changes, and cerebral atherosclerosis. *Acta Neurol. Scand. Suppl.* 102 1–94. 3887832

[B51] Sled J. G. Zijdenbos A. P. Evans A. C. (1998). A nonparametric method for automatic correction of intensity nonuniformity in MRI data. *IEEE Trans. Med. Imaging* 17 87–97. 9617910 10.1109/42.668698

[B52] Small S. A. Schobel S. A. Buxton R. B. Witter M. P. Barnes C. A. (2011). A pathophysiological framework of hippocampal dysfunction in ageing and disease. *Nat. Rev. Neurosci.* 12 585–601. 10.1038/nrn3085 21897434 PMC3312472

[B53] Styner M. Lieberman J. A. Pantazis D. Gerig G. (2004). Boundary and medial shape analysis of the hippocampus in schizophrenia. *Med. Image Anal.* 8 197–203. 10.1016/j.media.2004.06.004 15450215

[B54] Tamminga C. A. Stan A. D. Wagner A. D. (2010). The hippocampal formation in schizophrenia. *Am. J. Psychiatry* 167 1178–1193. 10.1176/appi.ajp.2010.09081187 20810471

[B55] Tepest R. Wang L. Miller M. I. Falkai P. Csernansky J. G. (2003). Hippocampal deformities in the unaffected siblings of schizophrenia subjects. *Biol. Psychiatry* 54 1234–1240. 14643091 10.1016/s0006-3223(03)00702-9

[B56] Velakoulis D. Wood S. J. Wong M. T. McGorry P. D. Yung A. Phillips L. (2006). Hippocampal and amygdala volumes according to psychosis stage and diagnosis: A magnetic resonance imaging study of chronic schizophrenia, first-episode psychosis, and ultra-high-risk individuals. *Arch. Gen. Psychiatry* 63 139–149. 10.1001/archpsyc.63.2.139 16461856

[B57] Vita A. De Peri L. Silenzi C. Dieci M. (2006). Brain morphology in first-episode schizophrenia: A meta-analysis of quantitative magnetic resonance imaging studies. *Schizophr. Res.* 82 75–88. 10.1016/j.schres.2005.11.004 16377156

[B58] Watson C. Andermann F. Gloor P. Jones-Gotman M. Peters T. Evans A. (1992). Anatomic basis of amygdaloid and hippocampal volume measurement by magnetic resonance imaging. *Neurology* 42 1743–1750. 10.1212/wnl.42.9.1743 1513464

[B59] Weickert T. W. Goldberg T. E. Gold J. M. Bigelow L. B. Egan M. F. Weinberger D. R. (2000). Cognitive impairments in patients with schizophrenia displaying preserved and compromised intellect. *Arch. Gen. Psychiatry* 57 907–913. 10.1001/archpsyc.57.9.907 10986554

[B60] Yang H. Yang S. Isen A. M. (2013). Positive affect improves working memory: Implications for controlled cognitive processing. *Cogn. Emot.* 27 474–482. 10.1080/02699931.2012.713325 22917664

[B61] Yoon T. Okada J. Jung M. W. Kim J. J. (2008). Prefrontal cortex and hippocampus subserve different components of working memory in rats. *Learn. Mem.* 15 97–105. 10.1101/lm.850808 18285468 PMC2275661

[B62] Zierhut K. C. Grassmann R. Kaufmann J. Steiner J. Bogerts B. Schiltz K. (2013). Hippocampal CA1 deformity is related to symptom severity and antipsychotic dosage in schizophrenia. *Brain* 136(Pt 3) 804–814. 10.1093/brain/aws335 23388407

